# Fish Rejections in the Marine Aquarium Trade: An Initial Case Study Raises Concern for Village-Based Fisheries

**DOI:** 10.1371/journal.pone.0151624

**Published:** 2016-03-10

**Authors:** Thane A. Militz, Jeff Kinch, Simon Foale, Paul C. Southgate

**Affiliations:** 1 Centre for Sustainable Tropical Fisheries and Aquaculture, College of Marine and Environmental Sciences, James Cook University, Townsville, Australia; 2 National Fisheries College, National Fisheries Authority, Kavieng, Papua New Guinea; 3 Centre for Tropical Biodiversity and Climate Change, College of Arts, Society & Education, James Cook University, Townsville, Australia; 4 Australian Centre for Pacific Islands Research and Faculty of Science, Health, Education and Engineering, University of the Sunshine Coast, Maroochydore, Australia; University of Minnesota, UNITED STATES

## Abstract

A major difficulty in managing wildlife trade is the reliance on trade data (rather than capture data) to monitor exploitation of wild populations. Collected organisms that die or are rejected before a point of sale often go unreported. For the global marine aquarium trade, identifying the loss of collected fish from rejection, prior to export, is a first step in assessing true collection levels. This study takes a detailed look at fish rejections by buyers before export using the Papua New Guinea marine aquarium fishery as a case study. Utilizing collection invoices detailing the species and quantity of fish (Actinopteri and Elasmobranchii) accepted or rejected by the exporting company it was determined that, over a six month period, 24.2% of the total fish catch reported (n = 13,886) was rejected. Of the ten most collected fish families, rejection frequency was highest for the Apogonidae (54.2%), Chaetodontidae (26.3%), and Acanthuridae (18.2%) and lowest for Labridae (6.6%) and Hemiscylliidae (0.7%). The most frequently cited reasons for rejection were fin damage (45.6% of cases), undersized fish (21.8%), and fish deemed too thin (11.1%). Despite fishers receiving feedback on invoices explaining rejections, there was no improvement in rejection frequencies over time (r = -0.33, P = 0.15) with weekly rejection frequencies being highly inconsistent (range: 2.8% to 79.4%; *s* = 16.3%). These findings suggest that export/import statistics can greatly underestimate collection for the marine aquarium trade as additional factors such as fisher discards, escapees, post-collection mortalities, and unregulated domestic trade would further contribute to this disparity.

## Introduction

Wildlife trade has evolved into a pivotal concern for both biodiversity conservation and sustainable development. The present century is afflicted with global declines in terrestrial and aquatic ecosystems, spurred by anthropogenic stressors and global climate change [1–3]. Exploitation associated with wildlife trade in the present era is a contentious issue. Sustainably managed wildlife trade can provide income for some of the least economically affluent people [4,5] though overexploitation of wildlife can be a principal cause of biodiversity loss [6,7]. Consequently, accurate monitoring of exploitation is critical.

An inherent flaw in managing the exploitation of wildlife trade is a reliance on trade data, and not capture data, as a proxy variable to monitor impacts of exploitation of wild populations [6]. The disparity between collection and trade is largely unknown for most wildlife trades. Collected organisms that die or are rejected before a point of sale often go unreported. Quantifying this unreported loss is a first step in correctly modelling and assessing the real impact of wildlife trade on wild populations.

Like much of the wildlife trade, the marine aquarium trade is largely characterized by international trade statistics [8–12]. This trade is responsible for the translocation of millions of marine organisms from their natural habitats to public and private aquaria worldwide [9]. Current proposals for more accurate monitoring of the marine aquarium trade suggest utilization of trade invoices as the way forward [11]. While this is likely to be the most feasible method for countries to monitor the industry, management recommendations made for source countries on the basis of trade data are hindered because trade may be unrepresentative of true collection levels. Prior to commercialized trade, fish may be lost due to fisher discards, quality control rejections by buyers, mortality, escape, and unregulated domestic trade, which can all accentuate the difference between numbers collected and traded. A secondary consequence of escapees, discards, and rejections leading to release is the risk of disease transmission, unnatural gene flow, and establishment of alien species populations [7,13,14]. A logical first step in addressing this potential issue is to assess the degree to which these factors may impact a fishery’s total catch.

Fish rejections are inevitable in the quality control process of supplying a trade largely built around aesthetics [9,15]. Where quality control falters, export of low quality fish can have negative repercussions for all operations in the region [16]. While buyer rejections of fish caught by fishers are known to occur within the trade, the proportion of the catch rejected from village-based fisheries has never been empirically evaluated beyond isolated collection events [17,18]. Village-based fisheries dominate the global supply of marine aquarium organisms, with most fish being derived from impoverished countries in the Indo-Western Pacific [9,11]. In this study we quantify the proportion of total catch rejected by buyers, evaluate reasons for rejection, and examine rejection frequencies over time using the entire Papua New Guinea (PNG) marine aquarium fishery as a case study.

## Materials and Methods

### Study Fishery

Papua New Guinea comprises the eastern part of the island of New Guinea and a number of smaller islands in the Indo-Western Pacific and is considered part of the Coral Triangle, a center of global marine diversity and a hot-spot of endemism. There is a rich tradition of fishing among the coastal and island communities of PNG. Signs of overexploitation of some commercially important marine income generating and food species have increased in recent times, especially in areas close to urban centers [19,20]. As an alternative livelihood option, the PNG National Fishery Authority (NFA) began expressing interest in the marine aquarium trade as early as 1990 [21]. However, no commercial action eventuated and it was not until 2007 that interest was reinvigorated when the NFA contracted a US-based consulting firm, EcoEZ Inc., to reassess marine resources with value to the marine aquarium trade. This consultancy subsequently developed into a three year project to develop a sustainable approach to a marine aquarium trade fishery with commercial realization to be achieved in the third year of the project [22].

Over the course of development, EcoEZ Inc. engaged eight communities in fish collection for the marine aquarium trade. These communities were all in the Central Province bordering the PNG capital of Port Moresby and included Fishermen Island, Roku, Pari, Gaire, Tarauama, Gabagaba, Keapara, and Kouderika. Six of these communities had their own demarcated Fishery Management Areas (FMAs) while the remaining two communities (Pari and Tarauama) had shared access to a single FMA. The FMAs defined the spatial unit in which all collection activities were to occur. Two of the FMAs, Fishermen Island and Keapara Village, were claimed to be certifiable under Marine Aquarium Council (MAC) standards [22,23].

All fishers were trained in collection and handling practices according to MAC certification standards [24,25] and there have been no reports of illegal fishing activity (i.e. use of chemicals or prohibited gear) occurring. Collection of aquarium fish was conducted using snorkel with a compliment of hand, fence, and barrier nets made from fine mesh. Fish were coerced into the fence or barrier nets by the presence of the fisher or with the aid of a ‘tickler’ stick at which point they were scooped up with hand nets and placed into submerged or floating holding containers. Fish were typically targeted as individuals or small groups. At the end of a fishing session, the fish were transported from the fishing grounds by canoe or motorized boat (generally < 10 km) to a holding enclosure, nets suspended from the surface or submerged containers, often close to shore and close to the fisher’s residence. Fish were held live within holding enclosures until purchased by a buyer from the exporting company.

### Data collection

Livestock collection operated with orders being given to fishers by the exporting company (EcoEZ Inc.) on a weekly basis. After several days of fishing, a buyer from the exporting company would visit fishers to purchase their catch. Purchasing was done by collating information on the catch and producing a collection invoice for the amount owed to fishers for their catch. Collection invoices detailed the identity and quantity of each fish species (Actinopteri and Elasmobranchii) accepted and/or rejected. Where fish were rejected the reason behind such rejections were often noted to provide fishers with feedback on their catch. This ‘catch-to-order’ method of fishery organization was perceived as a solution to avoid collecting species in excess of demand and to decrease the quantity of fish rejected by buyers; both problems known from the Indonesian and the Philippine fisheries [7,18,26].

The NFA retained an electronic copy of all collection invoices provided to them by EcoEZ Inc. as part of the contracted consultancy. However, records prior to 2010 are largely incomplete and a complete set of collection invoices could only be obtained for a six month period from January 1^st^ 2010 to June 14^th^ 2010 which was analyzed in this study (hereafter, the ‘study period’). Seven of the eight communities were engaged in fishing during the study period.

### Data Analysis

All data was transferred into Excel (Version 14). Collection invoices were taken at face value as misinformation could not be corrected for. However, corrections were made when species names were misspelled or listed with only a common name. Scientific names were matched with common names using an NFA-supplied EcoEZ Inc. identification guide. Validity of scientific names was confirmed using the World Register of Marine Species [27]. For purposes of analysis, all Apogonidae species were grouped as ‘Apogonidae spp.’ for two reasons: (1) the majority (55.9%) of apogonids could not be identified, accounting for 90.0% of all unidentified species, and (2) due to multiple species sharing the common name ‘yellow cardinal’ it is plausible those identified on collection invoices were in error. Fish were labelled as ‘unknown’ where neither a species name nor family could be assigned using the company’s identification guide.

At all levels of analysis, rejection frequencies were calculated by the number of fish rejected as a percentage of the total fish catch. In the case of explanations reported for rejections, responses deviated from definitive categories. In an iterative process, all explanations were grouped into eight encompassing categories (Table 1). The statistical package S-Plus (Version 8.0) was used to determine 95% confidence intervals for the fishery-wide rejection frequency using the Agresti-Coull method. Linear regressions were run in S-Plus comparing individual fishers’ rejection frequencies against their total catch and to compare rejection frequencies against their catch of two specific fish families. It was necessary to apply a square root transformation to fishers’ rejection frequencies to satisfy the assumptions of the linear regression analysis. All invoices were also time sorted by the week in which fishing commenced for a given collection invoice. Weeks were numbered chronologically from the start of the year. A Pearson’s correlation analysis was run using S-Plus to determine the nature of an association between time (i.e. fishing week) and rejection frequencies.

**Table 1:** Reasons given for fish rejections and the grouping terms used in this study.

As Grouped in this Study	Reported Reasons on Invoice
Too thin	Too thin
Undersized	Too small
Too fat	Too fat
Oversized	Too large
Not ordered	Wrongly Identified
	Not ordered
Body damage	Bruised
	Tissue damage
	Removed scales
	Bulging eye
Fin damage	Torn fin
Dead	Dead

## Results

### Fish Collections

**Fig 1:**
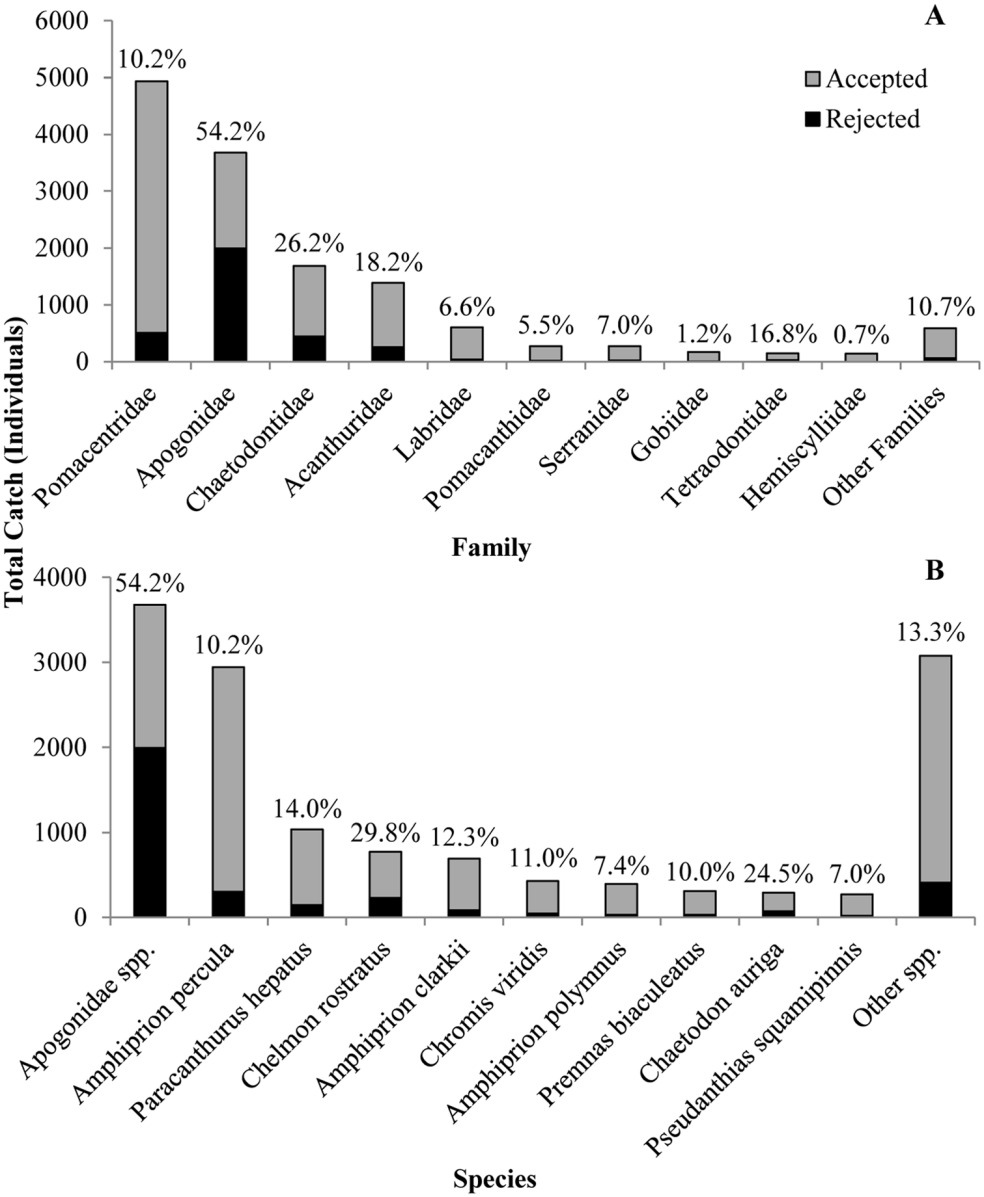
Total catch for the most collected fish families (A) and species (B) divided into those fish accepted and rejected by the exporting company. The percentage of catch rejected for a given family or species is presented as superscripts.

Collection invoices made available by the NFA show that a total of 13,892 fish were collected during the study period. Of these 83.6% could be identified to species whilst 99.9% were identified to family. The top ten collected families (of the 29 families identified) accounted for 95.8% of total fish collections while the top ten species (of the 134 species identified) collected accounted for 77.9% of total fish collections (Fig 1). Nearly all (98.5%) identified fish species collected were species known to be purchased by the exporting company.

**Fig 2:**
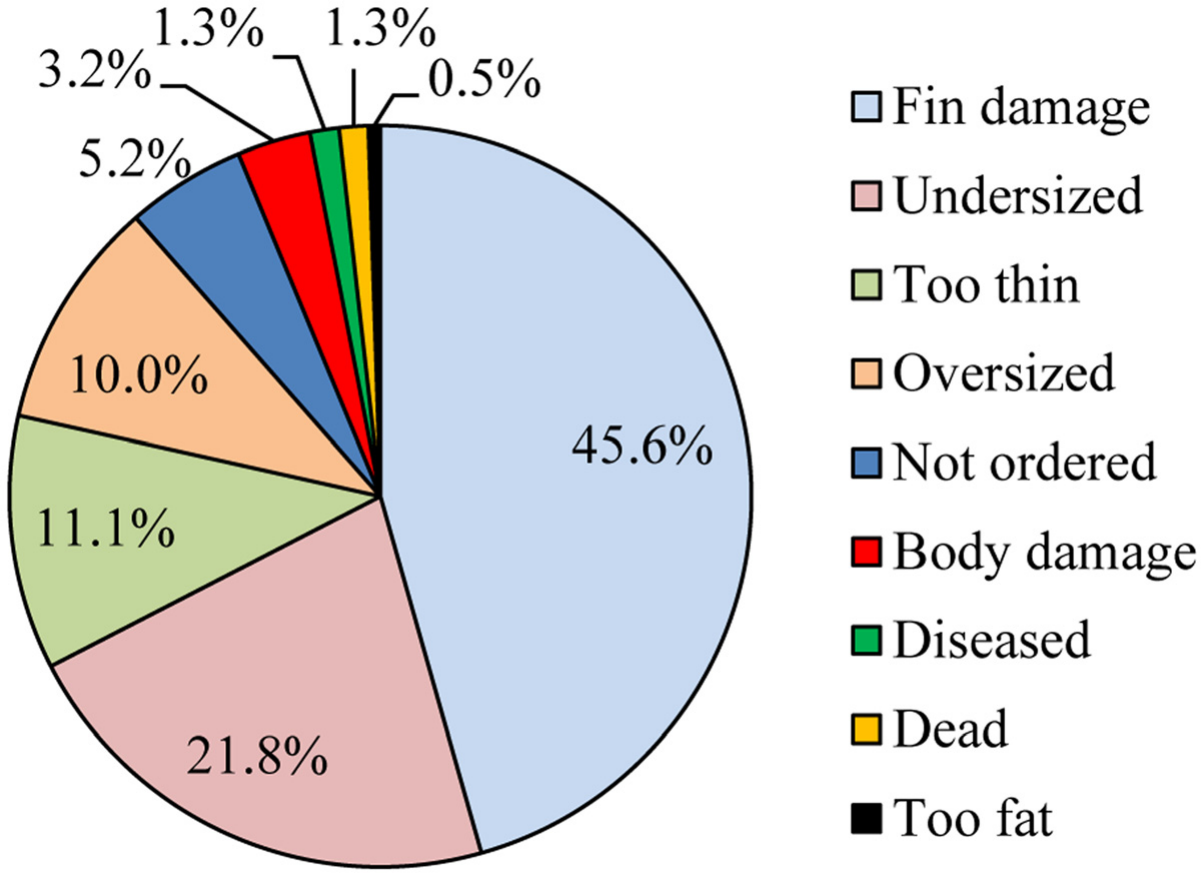
Reasons for rejecting individual fish as a percentage of explained rejections.

Across the entire fishery, 24.2% (95% confidence interval: 23.5 to 24.9%) of the total fish catch was rejected. Rejection frequencies among the most collected families ranged from 54.2% (Apogonidae) to as low as 0.7% (Hemiscylliidae; Fig 1). Of the top ten most collected species, the butterflyfish, *Chelmon rostratus*, had the highest rejection frequency (29.8%) while the anthias, *Pseudanthias squamipinnis*, had the lowest (7.0%; Fig 1). Explanations for rejection were recorded for 40.0% of all rejections (n = 3,360; Fig 2). The most common explanations were fish having fin damage (45.6%) or being undersized (21.8%). Chaetodontidae were particularly prone to fin damage, accounting for 48.6% of all fin damage cases (n = 613) and comprising 78.0% of all explained chaetodontid rejections (n = 382; Table 2). Rejections due to undersized fish were mostly associated with Pomacentridae, with this family accounting for 56.3% of all undersized catch (n = 293) and this factor account for 41.5% of explained pomacentrid rejections (n = 398; Table 2).

**Table 2:** Primary causes for rejection of fish from the five most collected families.

Family	Primary Causes for Rejection (% of explained rejections)
Pomacentridae	Undersized (41.5%)
	Fin damage (38.7%)
Apogonidae	Too thin (46.6%)
	Undersized (22.8%)
Chaetodontidae	Fin damage (78.0%)
	Undersized (14.4%)
Acanthuridae	Fin damage (40.8%)
	Oversized (38.5%)
Labridae	Fin damage (48.6%)
	Not ordered (18.9%)

### Fisher Performance

**Fig 3:**
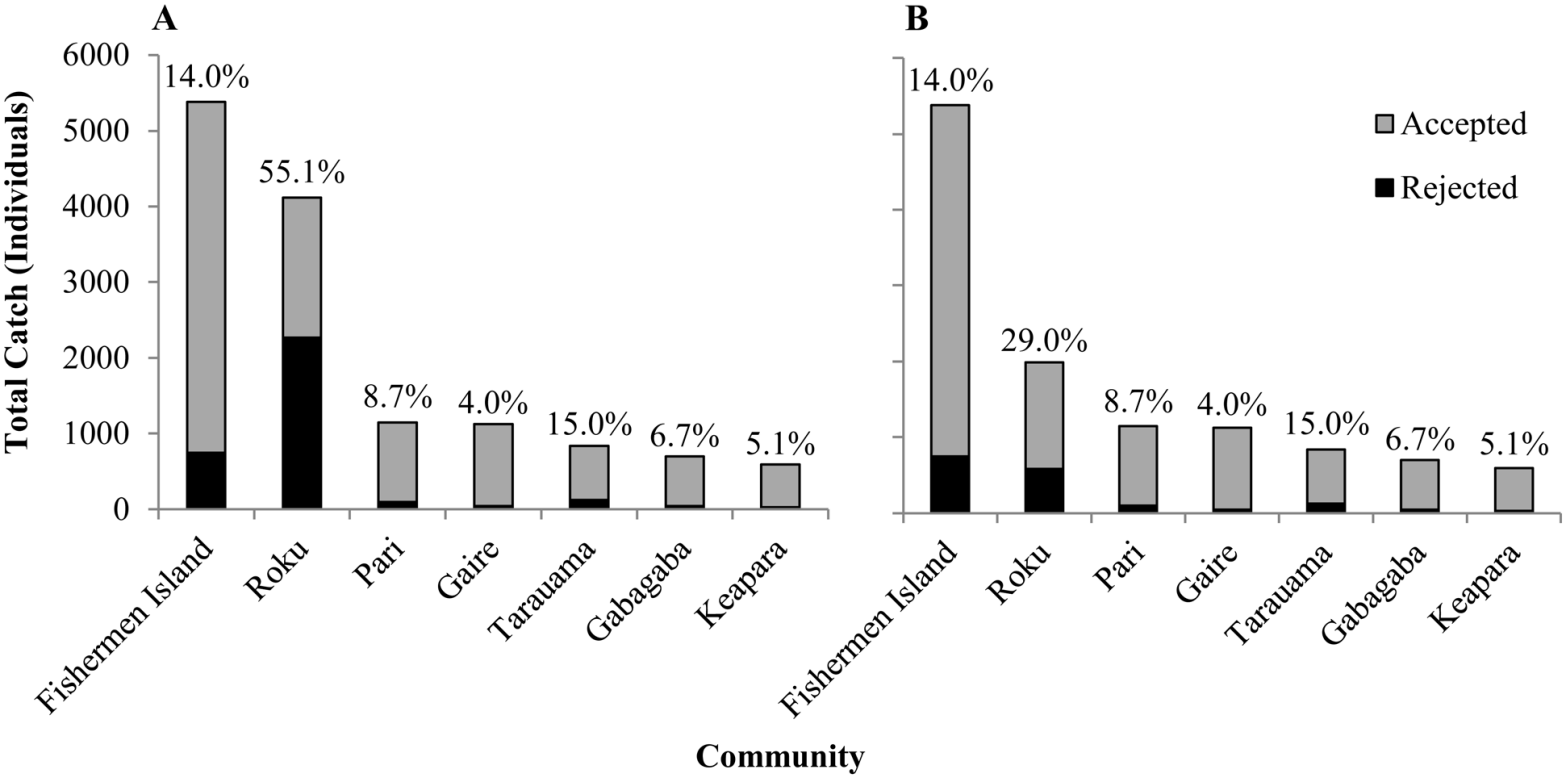
Total catch of fish by each community divided into those fish accepted and rejected by the exporting company. The percentage of total catch rejected is presented as superscripts. (A) Presents all data during the study period. (B) Data with one week of fishing at Roku omitted.

From the total catch, 91.0% could be attributed to individual fishers; the remaining catch resulted from multiple fishers collaborating or unidentified fishers. A single community, Roku, was responsible for 67.3% of all rejections despite contributing only 17.6% of all accepted fish (Fig 3). One week of fishing at Roku over the study period contributed to 50.1% of all rejections in the fishery over the study period. However, even when this week is removed from the data set, Roku still had the highest rejection frequency (29.0% of total catch), which was nearly double that of the village with the next highest rejection frequency (Tarauama: 15.0%; Fig 3).

**Fig 4:**
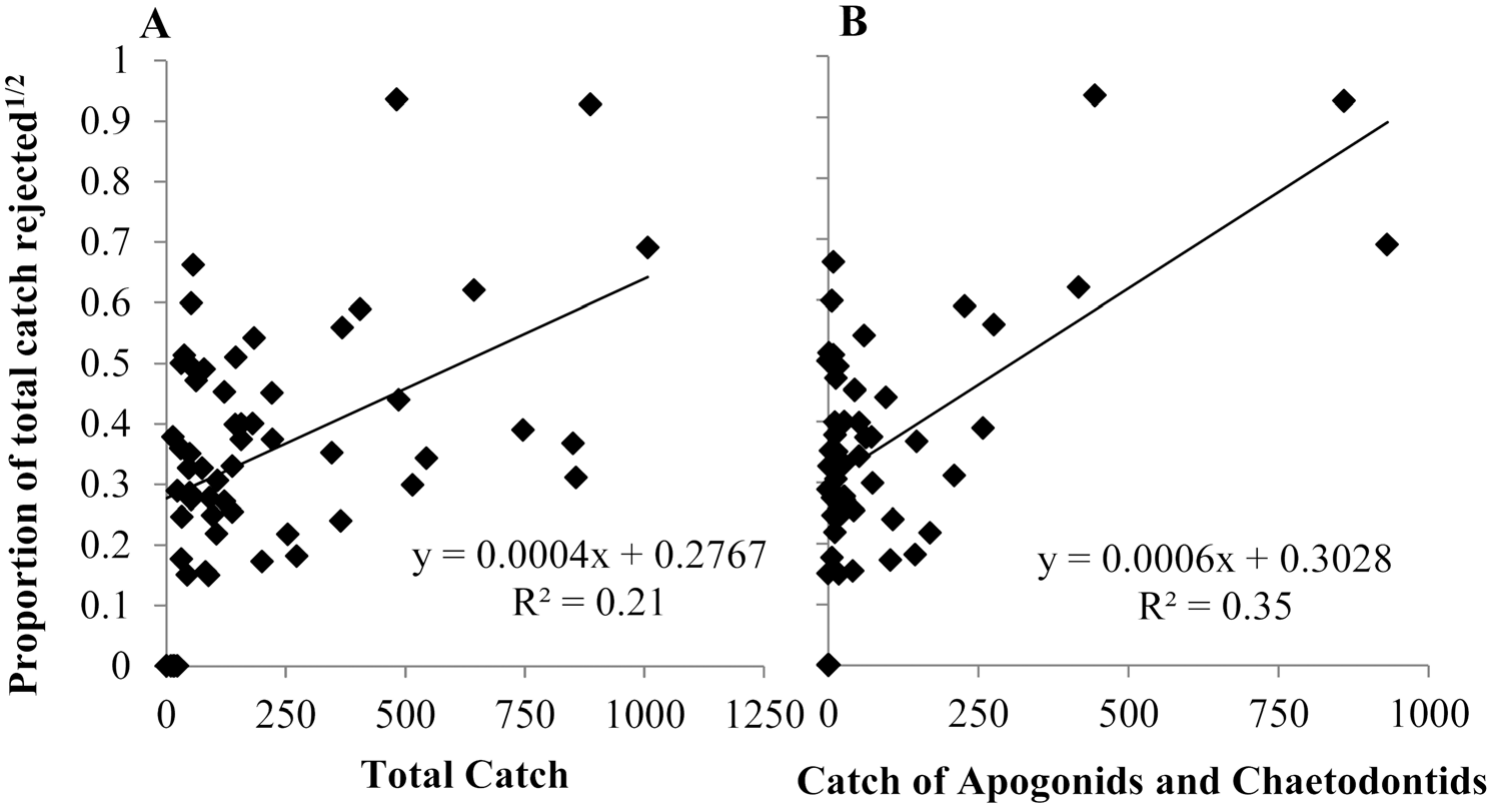
Proportion of total catch rejected (square root transformed) plotted against (A) total catch and (B) catch of apogonids and chaetodontids over the study period for individual fishers.

The high percentage of rejections at Roku is due to the targeted fish species, with two-thirds (66.1%) of the accepted catch (n = 1848) from this village being composed of Apogonidae and Chaetodontidae, the highest of any village. These two families of fish accounted for 72.5% of all rejections in the fishery. There was a significant positive relationship between rejection frequency and a fisher’s catch of Apogonidae and Chaetodontidae fishes (*F*_1,56_ = 29.55, P <0.001; Fig 4). The relationship between fishers’ rejection frequencies and their catch of Apogonidae and Chaetodontidae explained almost twice the total variation (R^2^ = 0.35) in rejection frequencies than a relationship with total catch of all fish species (R^2^ = 0.21, *F*_1,56_ = 15.22, P <0.001; Fig 4). There was no significant correlation between time (i.e. fishing week) and rejection frequency (r = -0.33, *t*_(2)18_ = -1.49, P = 0.15) with notable inconsistencies in rejection frequencies between weeks (range: 2.8% to 79.4%; *s* = 16.3%).

## Discussion

Characterization of the marine aquarium trade is dominated by international trade statistics [8–12] with proposals for improved monitoring to utilize trade invoices [11]. While this is likely to be the most feasible method to monitor relative exploitation by the trade, management recommendations made on the basis of export/import data must account for their limitations. Here we show that a minimum of 24.2% of the total fish catch from the PNG marine aquarium fishery went unreported in export/import invoices during the six month study period. This unreported catch consisted only of rejections at the collection level. The actual difference between collection and export is likely to be even greater when mortalities at the exporting facility and unreported domestic trade are factored in. Those species and villages with the highest rejection frequencies indicate where management considerations are needed most.

### Fisher Performance

Despite being a relatively new fishery, it is unlikely that fisher inexperience contributed to the results because fishers had already collected a minimum of 31,365 organisms prior to the study period in 2008–2009 [12]. Further, fishers who collected more fish (i.e. had more experience) tended to have higher, rather than lower, rejection frequencies. Widespread use of inappropriate collection techniques can also be ruled out as a factor contributing to rejections as all fishers were trained according to the MAC certification standards [25].

Competition and rivalry between fisher groups targeting aquarium organisms has been reported in Indonesia [4]. Where competition for shared resources occurs, resource depletion can be accelerated through a ‘tragedy-of-the-commons’ scenario [28], albeit within a collectively owned territory. Interestingly, the shared FMA of Pari and Tarauama did not result in exceptionally high rejection frequencies for either community that would be expected from such a scenario. Rather, the species composition of catches seemed to explain the majority of variation in rejection frequencies for fishers.

Rejections translate to a reduction in sale-per-unit-effort for the fishers as the buyers would only compensate fishers for accepted fish. This method of purchasing catch is commonplace in the trade [4,17,18,29]. The wasted effort in collecting/holding a fish that becomes rejected, however, is not perceived as a disincentive to avoid rejections in the future [18,26,30]. We are not suggesting fishers collected fish indiscriminately as nearly all collected species identified were known to be purchased by the exporter. Rather, the potential for financial gain, coupled with the effort required to isolate a fish for discarding, incentivizes retention of fish even if there is only a slight chance of purchase by buyers. For example, in the case of rejections attributed to damaged fish (the most common reason for rejections), it is most likely that damage occurred during collection and/or subsequent handling after the fisher correctly identified suitable species. Damage therefore would only be realized after the point of capture. This presents the fisher with the decision to either discard the fish, potentially benefiting the long term productivity of the fishery (but risk someone else catching it), or retain the fish with the chance that a buyer may accept it. Only the latter option offers potential for an immediate economic return; a strong incentive for fishers in low-income countries like PNG [31–33].

Further, the primary reason for buyers to itemize rejected fish and give an explanation for rejection on collection invoices was to provide fishers with continual feedback about their catch. Despite provision of this feedback, fisher performance failed to improve over the duration of the study period. These scenarios highlight how education alone may not achieve alterations in behavior and suggest that economic incentives/disincentives must be coupled with education to promote change in practice. A reward system such as third party eco-certification [34], where buyers are incentivized to reward fishers and/or villages for low rejection frequencies, may have merit in these circumstances.

### Reasons for Rejections

Rejection frequencies varied greatly between species and even between families being collected. This limits the capacity for a single rejection value to accurately represent the disparity between collection and export/import statistics. Those families most frequently rejected (i.e. Apogonidae, Chaetodontidae, Acanthuridae) are where the greatest inaccuracies between collection and export/import statistics are likely to occur.

Fin damage was the most cited reason for rejection and a primary cause of rejections for four of the five most collected families. This suggests mitigation of fin damage be made a management priority for the PNG fishery. Predictably, those families characterized by more elaborate and delicate fins (i.e. Chaetodontidae and Acanthuridae) had a greater proportion of rejections attributed to fin damage than families with smaller, compact bodies (i.e. Pomacentridae and Apogonidae). Such damage likely arises from collection where abrasion from netting material damages the soft tissue of the fins or through handling/aggression from co-habiting fish after capture [26,30]. Ensuring fishers use appropriately sized netting (3–28 mm depending on target fish [29]) for collection and isolating aggressive fish during holding would be first steps in reducing the frequency of fin damage rejections.

Oversized and undersized fish are another cause of rejection that occurred at the point of collection. Of the top five fish families, undersized fish were the most cited cause of rejection only for the Pomacentridae. Given the high demand for certain pomacentrids (namely *Amphiprion percula*) fishers may be tempted to collect potentially undersized fish in an attempt to fill orders and increase economic returns [5,26]. Aquarium fishers in Indonesia knowingly collected undersized *Amphiprion* spp. given their ease of capture in the hope that a buyer would accept a portion of the undersized catch [5,18].

The same logic likely applies to the collection of oversized fish, a primary cause of rejection for the Acanthuridae. These fishes have asymptotic growth curves [35] with juvenile fish being sought by the aquarium trade [9,17]. Where juveniles are limited in supply on the reef due to seasonal recruitment [36,37] fishers may be tempted to collect oversized fish. Additionally, Acanthuridae are a target food species in PNG with many aquarium fishers simultaneously engaged in subsistence fishing for income [38]. This suggests that oversized fish may represent catch from food fishing where fishers try to obtain a higher price from aquarium trade buyers before sale at local markets or consumption [5,9].

Fish rejected for being too thin (i.e. Apogonidae), in contrast, is likely to be a consequence of post-capture care during holding where fish are not fed or fed inappropriately resulting in emaciation. A reduction of holding times through more frequent visitation to fishers by buyers, and ensuring buyers are equipping fishers with appropriate fish feeds and knowledge on fish husbandry practices, would help ensure such rejections are minimized.

### Ecological Consequences

The vast majority of explained rejections in this study involved live fish. The end fate of such fish is uncertain. The most likely scenarios are that rejected fish are returned to the sea, held for an alternative buyer, held until aesthetic impairment improves (for fin damage/body damage cases), eaten, or used as bait in subsistence food fishing. In the case of the PNG fishery, there were no alternative buyers and fishers did not have facilities for holding fish beyond a couple days. This would suggest rejected fish did not find their way into the trade through an alternative route.

Where post-collection rejections result in return of live fish to the sea, either for the purpose of survival or as escaped bait, serious ramifications can result. Rejection due to identifiable disease or body damage (possibly caused by disease) accounted for 4.5% of explained rejections. Pathogenic organisms are known to proliferate under tight holding conditions and with host stress [39,40], both of which are inherent in the collecting process. Thus, the release of any rejected fish confined for a period of time poses the risk of releasing a fish with amplified levels of disease [13]. Where fish are released in an area far removed from the point of collection, release can also result in unnatural gene flow and establishment of alien species [7,13]. This has previously been documented in Indonesia where rejections of the Banggai Cardinalfish, *Pterapogon kauderni*, along the aquarium supply chain have resulted in the establishment of alien populations [41,42]. These risks merit consideration by the regulatory authorities of supply countries and protocols deemed appropriate for dealing with rejected fish communicated to buyers and fishers. Discarding fish likely to be rejected immediately after capture would be the surest way to prevent such risks but places the onus on the fisher to accurately gauge fish of acceptable quality, the socio-economic complications and potential management of which have already been addressed above.

In addition to these risks, the survival of aquarium fishery releases are unknown and difficult to assess [43]. Average release mortality from a meta-analysis of recreational sport fishing show a mean mortality of 18% across studied species with post-release predation potentially accounting for a further 20% loss [44]. However, the differences in gear used and the biology, ecology, and life history of the target species, limit the applicability of such data to aquarium fisheries. The majority of fish collected in this study and traded globally (i.e. Pomacentridae and Apogonidae) [9,11] are known to have adult home ranges of a few meters or less [45–47]. Releasing these fishes outside of their home range in different habitats may limit their chances of survival [48]. This is expected to be true for habitat dependent fishes like clownfish (Amphiprioninae) which are reliant on host anemones for survival [46]. In reality, it is unlikely that many of the rejected fish are returned to their reef of origin. In Indonesia, live rejected fish were thrown back into the sea behind the buyer’s facility regardless of origin [18] and this was likewise noted for rejected coral collected for the aquarium trade [4]. Until estimates of the proportion of rejected fish returned to sea and their survival are known, it would be advisable that fishery management decisions be made following the precautionary principle and assume rejections are lost from natural populations. To accurately gauge these risks and loss it may be more prudent for fishery management agencies to begin developing policies to monitor collection records rather than trade invoices for species suspected of frequent rejections.

### Rejection Frequency in the Trade

The majority of supply to the marine aquarium trade originates from impoverished countries of similar economic status to PNG [9,11] and, where fishers face economic pressures, unsustainable practices can flourish [17,26,29]. Operated as ‘fish-to-order’ with direct supply (i.e. the fisher directly supplies the exporting company) and fishers trained to MAC standards, rejections should have been minimized within the PNG fishery. However, rejection frequencies reported for PNG are higher than any previously reported figure. Prior to this study, quantitative evaluations of rejection frequencies have been limited to brief accounts on collections. Kinch [17] reported an exporter rejection frequency of 11.6% (n = 493) for fish collections from Rarumana, Solomon Islands over a four day period in 2004; however the representativeness of this sample is uncertain given the inconsistencies in quality noted by the buyer. Such inconsistencies were also noted in the PNG fishery with weekly rejection frequencies ranging from 2.8% to 79.4% during the study period.

Where middlemen operate in the market chain that facilitate collection from fishers and then on-sell to exporters [4,9], quality assessment and rejection occurs on multiple levels within the supply chain [49]. Where the MAC [18] looked at fish rejection frequencies in Indonesia during supervised fishing trips they found buyers rejected 5.7% (n = 5,052) and 1.4% (n = 14,246) during an eight and six day fishing trip, respectively. However, these rejection frequencies were considered low by the collectors and likely only represent preliminary rejections as buyers would then on-sell the catch to an exporter were further, unreported, rejections occur [26]. Such supply networks can also limit feedback to fishers on the quality of their catch and what the end market is demanding.

The high rejection frequency reported in this study results from the six month study period allowing the reported inconsistencies to be averaged out. The methodology employed in this study to analyze data routinely collected by buyers would also have negated potential observer effects on fisher performance present in previous studies. It is likely that the rejection frequency observed within the PNG fishery is representative of village-based fishing operations in developing countries. Many island nations with marine aquarium fisheries have only a single exporting company [50], eliminating the possibility that rejected fish enter the market through an alternative buyer. Even in Indonesia and the Philippines, where multiple buyers operate in close proximity, fishers generally deal exclusively with a single buyer with strong fisher-buyer relationships being driven by financial and social pressures [4,18]. Other aquarium fisheries are also burdened with excessive transportation distances between fishing grounds and place of sale, exceeding hundreds of kilometers [17,18], suggesting fish are exposed to even greater levels of transport stress than in the current study. However, until comparable data from other marine aquarium fisheries is made available, the authors caution overextrapolation of the results from this case study. Much lower levels of rejection would be expected in fisheries operating in affluent countries or where fishers are directly employed by the exporting company (or self-employed) allowing for increased feedback on catch. In Hawaii, for example, where collectors are typically self-employed, collection discards and mortality following capture were < 1% of total catch for November 2008 [51]. Quality of the catch was assessed onsite immediately after capture with discards being returned to the sea and no further rejections were reported to occur before sale.

## Conclusions

While global monitoring of trade through catch data continues to lack feasibility, case studies demonstrating the difference between exports and collections are needed to translate trade statistics into more accurate representations of fishery catch. Identifying the loss of collected fish through buyer rejections prior to export is one key component in the difference between collection and export statistics. Application of rejection frequencies as a correction tool and undertaking localized studies, as done here, can ground truth in the underestimation of catch and provide a better estimate of overall mortality for aquarium fisheries from trade invoices. In this initial case study, a rejection frequency of one in every four collected fish raises concern about the quantity of unreported catch that may be occurring in other aquarium fisheries. Such concern is further compounded because rejections are one of several factors contributing to the disparity between catch and export statistics. Further research effort aimed at addressing mortality along supply chains and domestic trade within supply countries, would greatly aid determination of the true fishing effort from trade data.
